# A Structural Equation Modeling Approach to Meta-analytic Mediation Analysis Using Individual Participant Data: Testing Protective Behavioral Strategies as a Mediator of Brief Motivational Intervention Effects on Alcohol-Related Problems

**DOI:** 10.1007/s11121-021-01318-4

**Published:** 2021-11-12

**Authors:** David Huh, Xiaoyin Li, Zhengyang Zhou, Scott T. Walters, Scott A. Baldwin, Zhengqi Tan, Mary E. Larimer, Eun-Young Mun

**Affiliations:** 1 School of Social Work, University of Washington, 4101 15th Ave NE, Box 354900, Seattle, WA 98105-6299 USA; 2 Department of Health Behavior and Health Systems, University of North Texas Health Science Center, Fort Worth, TX USA; 3 Department of Biostatistics and Epidemiology, University of North Texas Health Science Center, Fort Worth, TX USA; 4 Department of Psychology, Brigham Young University, Provo, UT USA; 5 Department of Psychiatry and Behavioral Sciences, University of Washington, Seattle, WA USA

**Keywords:** Complex synthesis, Integrative data analysis, Indirect effect, Bootstrap inference with multiple imputation, Brief alcohol intervention

## Abstract

This paper introduces a meta-analytic mediation analysis approach for individual participant data (IPD) from multiple studies. Mediation analysis evaluates whether the effectiveness of an intervention on health outcomes occurs because of change in a key behavior targeted by the intervention. However, individual trials are often statistically underpowered to test mediation hypotheses. Existing approaches for evaluating mediation in the meta-analytic context are limited by their reliance on aggregate data; thus, findings may be confounded with study-level differences unrelated to the pathway of interest. To overcome the limitations of existing meta-analytic mediation approaches, we used a one-stage estimation approach using structural equation modeling (SEM) to combine IPD from multiple studies for mediation analysis. This approach (1) accounts for the clustering of participants within studies, (2) accommodates missing data via multiple imputation, and (3) allows valid inferences about the indirect (i.e., mediated) effects via bootstrapped confidence intervals. We used data (*N* = 3691 from 10 studies) from Project INTEGRATE (Mun et al. *Psychology of Addictive Behaviors*, *29*, 34–48, 2015) to illustrate the SEM approach to meta-analytic mediation analysis by testing whether improvements in the use of protective behavioral strategies mediate the effectiveness of brief motivational interventions for alcohol-related problems among college students. To facilitate the application of the methodology, we provide annotated computer code in R and data for replication. At a substantive level, stand-alone personalized feedback interventions reduced alcohol-related problems via greater use of protective behavioral strategies; however, the net-mediated effect across strategies was small in size, on average.

**Supplementary Information:**

The online version contains supplementary material available at 10.1007/s11121-021-01318-4.

## Introduction

Mediation analysis is used to evaluate whether the effects of an intervention on health outcomes occur because of change in a key behavior targeted by the intervention. Most of the existing methodological research and applications of mediation analysis have focused on individual studies. However, beyond assessing the overall effectiveness of a treatment, single-study intervention trials are frequently underpowered to evaluate pathways of change (Fritz et al., 2015). A meta-analytic approach to mediation analysis that leverages data from multiple studies provides an opportunity to test pathways of change with greater statistical power. However, the literature showing how to conduct mediation analysis in a meta-analytic context has been limited to aggregate data (Cheung & Chan, 2005). This paper focuses on methods for conducting mediation analysis using individual participant data (IPD) from multiple studies.

The most widely used method for combining data from multiple studies is meta-analysis using study-level, aggregate data (e.g., means, *SD*s, correlations); however, standard meta-analysis methods either do not lend themselves to mediation testing or do not accommodate IPD from multiple studies. For example, meta-regression is used to examine moderators of intervention effects—study-level predictors that are associated with the size of the effect—not mediation. In contrast, newer approaches using aggregate data, such as meta-analytic structural equation modeling (MASEM), can provide a test of mediation (i.e., indirect effects) when pooling data from multiple studies (Cheung, 2014, 2015). Correlation-based MASEM is a prevailing approach to meta-analytic mediation analysis in which correlation or covariance matrices extracted from published reports or generated from the raw data (Cheung & Chan, 2005, 2009) are combined to create a pooled correlation or covariance matrix that is subsequently analyzed using structural equation modeling (SEM; e.g., Wilson et al., 2016). Effect sizes and standard errors may also be utilized to test mediated effects via marginal likelihood synthesis, sequential Bayesian methods, or parameter-based MASEM (see van Zundert & Miočević, 2020 for a comparison).

However, because prevailing approaches for meta-analytic mediation analysis typically rely on aggregate data extracted from published reports, the findings may be confounded with study-level differences that are unrelated to the mechanism of interest. For example, Riley et al. (2010) illustrated a meta-regression of ten clinical trials for hypertension where the estimated treatment effect was smaller in men compared to women, whereas a one-step IPD meta-analysis that examined participant-level information directly within studies did not support a clinically significant difference in treatment effect by sex. The apparent superiority of treatment with women was an artifact of studies with larger proportions of female participants tending towards larger effect sizes, though for reasons unrelated to sex. Specifically, when treatment effects by sex were evaluated within studies, the differences in treatment response were not clinically significant. Consequently, the study-level summaries that are commonly utilized can make these approaches more prone to ecological inference bias. An advantage of MASEM is that within-study variables (e.g., sex) can be included in the model, which can avoid introducing ecological biases when individual-level data are aggregated and analyzed as study-level data (e.g., proportion of females in the study); however, this generally requires access to raw IPD.

A limitation of correlations as the input data for a mediation analysis is the loss of scale-level information since correlation coefficients are standardized within each study to have a mean of zero and a standard deviation of one. This allows the pooling and comparison of the correlations across studies but assumes that the bivariate correlations correspond with the same range of values on the variable scales across intervention groups and levels of the outcome and mediator variables within studies. In practice, it is difficult to know whether these assumptions are reasonable without verifying them with IPD. If these assumptions are not met, then the resulting inference could be biased.

Furthermore, MASEM and existing approaches utilizing aggregate data are generally limited by the information disclosed in intervention reports, which frequently do not include all outcomes that were assessed (see Mun et al., 2021), let alone correlations among key variables of interest. Thus, MASEM and other mediation modeling approaches that rely on aggregate data may not be possible in many cases without access to IPD or unreported aggregate data. Finally, with only aggregate data, it is impossible to check and verify whether the original data were appropriately analyzed and reported (e.g., the assumptions of multivariate normal distribution, data that is missing at random).

Meta-analysis using IPD provides an opportunity to more rigorously evaluate the pathways by which treatments improve health outcomes at the individual level. Furthermore, a mediation analysis with IPD permits a longitudinal analysis that controls for baseline levels of (a) the mediator, (b) the outcome, and (c) any relevant covariates.

The current paper proposes an SEM approach using IPD that (a) accounts for the clustering of participants within studies, (b) accommodates missing data via multiple imputation, and (c) allows valid inferences about the indirect effect (i.e., mediated effect) via bootstrapped confidence intervals in an integrative data analysis (IDA) that estimates the entire model in one step, after previously establishing commensurate measures (see Hussong et al., 2013 for typical considerations for IDA). In this article, we first introduce the motivating research question and example data. Second, we outline a meta-analytic mediation modeling approach that can accommodate the clustered data structure of participants nested within studies. Third, we discuss how to estimate confidence intervals for the indirect and total effects of intervention for the purpose of statistical inference. Finally, we illustrate the meta-analytic mediation analysis using data drawn from Project INTEGRATE (Mun et al., 2015) and discuss the implications of our method for both methodological and substantive research.

The motivating research question is whether improvements in protective behavioral strategies (PBS) mediate the effectiveness of brief motivational interventions for alcohol-related problems among college students who drink. PBS are specific cognitive-behavioral strategies that can be used prior to or during alcohol consumption to reduce alcohol-related problems (Martens et al., 2013). In the past two decades, promoting the use of PBS has become a common component of interventions for reducing alcohol-related problems among college drinkers (Ray et al., 2014). However, there has been mixed evidence on the extent to which improvements in PBS can explain the effect of brief motivational interventions on reducing alcohol use and related problems, with most evidence coming from cross-sectional data (Reid & Carey, 2015). We detail a longitudinal mediation analysis approach to evaluate whether improvements in PBS following brief motivational intervention are associated with subsequent reductions in alcohol-related problems among college students who drink.

## Motivating Data: The Project INTEGRATE Study

The motivating data are drawn from Project INTEGRATE, a large-scale IPD meta-analysis project evaluating brief motivational interventions for college drinking across 24 independent intervention studies (Mun et al., 2015). From the Project INTEGRATE data set, we selected ten studies that were randomized controlled trials assessing PBS and alcohol-related problems at baseline and at least one post-baseline assessment. Participants in the included studies were randomized to a control group or one of three brief motivational interventions: (1) individually delivered motivational interviewing with personalized feedback (MI + PF), (2) stand-alone personalized feedback (PF), or (3) group-based motivational interviewing (GMI). Because PBS is not applicable for non-drinkers, we only included participants within each study who reported at least one drink in the past 1 or 3 months, depending on the study, at post-baseline assessment. Table 1 summarizes the intervention arms and corresponding sample sizes for the combined sample of drinkers from the ten studies that met the study inclusion criteria. Eight of the 10 studies were two-arm trials that evaluated a single brief motivational intervention, whereas studies 9 and 21 evaluated two or more intervention groups.

**Table 1:** The combined sample by intervention group and study

**Study**	**Intervention group (*n*)**				**Follow-up** **(in months)**	**Reference**
	**Control**	**MI + PF**	**PF**	**GMI**		
2	74	–	70	–	2	White et al. (2008)
8a	429	–	398	–	12	Larimer et al. (2007)
8b	585	–	544	–	12	Larimer et al. (2007)
8c	131	–	113	–	12	Larimer et al. (2007)
9	78	79	77	80	3	Lee et al. (2009)
12	81	76	–	–	1	Wood et al. (2007)
16	86	–	–	97	1	LaBrie et al. (2009)
18	67	–	73	–	1	Martens et al. (2010)
21	66	68	59	–	3	Walters et al. (2009)
22	189	171	–	–	12	Wood et al. (2010)
**All**	1786	394	1334	177		

The follow-up (in months) is the first post-baseline assessment for which both mediation and outcome data were collected in the study.

*MI* + *PF* individually delivered motivational interviewing intervention with personalized feedback, *PF* stand-alone personalized feedback intervention, *GMI* group motivational interviewing intervention

The mediator variable, PBS, was measured using five different scales across the original studies, which were subsequently harmonized and made commensurate by using a generalized partial credit model (Muraki, 1992), which is an extension of the hierarchical two-parameter logistic item response theory (2-PL IRT) model that we reported for alcohol-related problems (Huo et al., 2015). The measurement work to establish PBS trait scores can be found in Mun et al. (2015, 2016). With respect to the motivating data, studies 2, 8a, 8b, 8c, and 9 used the 10-item Protective Behavioral Strategies (PBS; American College Health Association, 2001) measure; studies 16, 18, and 21 used the 15-item Protective Behavioral Strategies Scale (PBSS; Martens et al., 2005); and studies 12 and 22 used the seven-item Drinking Restraining Strategies (DRS; Wood et al., 2007) measure. Study 22 incorporated an additional nine-item measure asking about Drinking Strategies. These scales shared similarly worded items, from which five collapsed items across scales provided overlap across studies when estimating item parameters.

The outcome variable, alcohol-related problems, was assessed using six different scales across the original studies. We used latent trait scale scores estimated from hierarchical, 2-PL IRT models for multiple groups to establish commensurate alcohol-related problems trait scores for all participants across studies and time (Huo et al., 2015; Mun et al., 2015). With respect to the motivating data, studies 2, 8a, 8b, 8c, 9, 16, and 21 used the Rutgers Alcohol Problem Index (RAPI; White & Labouvie, 1989); studies 8a, 8b, 8c, 9, 12, 16, and 22 used the Young Adult Alcohol Problems Screening Test (YAAPST; Hurlbut & Sher, 1992); study 12 also used the Alcohol Dependence Scale (Skinner & Allen, 1982; Skinner & Horn, 1984); study 18 used the Brief Young Adult Alcohol Consequences Questionnaire (BYAACQ; Kahler et al., 2005); and study 21 used the Alcohol Use Disorders Identification Test (AUDIT; Saunders et al., 1993). For readers interested in the technical details regarding how the measures of PBS and alcohol problems used in the motivating data were made commensurate, the harmonization work is discussed extensively in earlier reports (Huo et al., 2015; Mun et al., 2015, 2016, 2019).

The sample for the present analysis included a total of 3691 students, with approximately two-thirds (63.8%) female. Most of the students identified as White (78.3%), and just over half of the participants (56.2%) were first-year or incoming college students. Table 2 provides a descriptive summary of all variables, including rates of missing data, by study and time point.

**Table 2:** Participant characteristics, proportions of missing data, and sample sizes by study

	**Study**										
	**2** (*n* = 144)	**8a** (*n* = 827)	**8b** (*n* = 1129)	**8c** (*n* = 244)	**9** (*n* = 314)	**12** (*n* = 157)	**16** (*n* = 183)	**18** (*n* = 140)	**21** (*n* = 193)	**22** (*n* = 360)	**All studies** (*N* = 3691)
**Sex**											
- Female	44 (30.6%)	576 (69.6%)	688 (60.9%)	147 (60.2%)	200 (63.7%)	83 (52.9%)	183 (100%)	101 (72.1%)	125 (64.8%)	207 (57.5%)	2354 (63.8%)
- Male	100 (69.4%)	251 (30.4%)	441 (39.1%)	97 (39.8%)	114 (36.3%)	74 (47.1%)	-	38 (27.1%)	68 (35.2%)	153 (42.5%)	1336 (36.2%)
- Missing	-	-	-	-	-	-	-	1 (0.7%)	-	-	1 (0.03%)
**Race**											
- White	101 (70.1%)	713 (86.2%)	752 (66.6%)	210 (86.1%)	230 (73.2%)	143 (91.1%)	118 (64.5%)	127 (90.7%)	166 (86.0%)	330 (91.7%)	2890 (78.3%)
- Non-White	40 (27.8%)	103 (12.5%)	369 (32.7%)	31 (12.7%)	84 (26.8%)	11 (7.0%)	63 (34.4%)	13 (9.3%)	27 (14.0%)	30 (8.3%)	771 (20.9%)
- Missing	3 (2.1%)	11 (1.3%)	8 (0.7%)	8 (1.2%)	-	3 (1.9%)	2 (1.1%)	-	-	-	30 (0.8%)
**First-year student**											
- No	59 (41.0%)	435 (52.6%)	592 (52.4%)	152 (62.3%)	-	152 (96.8%)	-	98 (70.0%)	115 (59.6%)	-	1603 (43.4%)
- Yes	85 (59.0%)	386 (46.7%)	532 (47.1%)	91 (37.3%)	314 (100%)	5 (3.2%)	183 (100%)	41 (29.3%)	78 (40.4%)	360 (100%)	2075 (56.2%)
- Missing	-	6 (0.7%)	5 (0.4%)	1 (0.4%)	-	-	-	1 (0.7%)	-	-	13 (0.4%)
**Alcohol-related problems**											
** - Baseline**											
- Mean (*SD*)	−0.5 (0.7)	0.1 (0.9)	0.2 (1.0)	0.1 (0.9)	0.9 (0.7)	0.4 (0.6)	−0.1 (0.9)	0.1 (0.9)	0.9 (0.8)	0.0 (0.9)	0.2 (0.9)
- Range	[−1.7, 1.2]	[−1.5, 3.5]	[−1.7, 3.5]	[−1.5, 2.6]	[−1.2, 2.9]	[−1.2, 1.8]	[−1.7, 1.9]	[−1.6, 2.1]	[−0.7, 2.9]	[−1.5, 2.0]	[−1.7, 3.5]
- Missing	-	3 (0.4%)	3 (0.3%)	-	-	-	-	-	-	-	6 (0.2%)
** - Post-baseline**											
- Mean (*SD*)	−0.7 (0.7)	0.2 (0.8)	0.3 (0.9)	0.2 (0.9)	0.8 (0.8)	0.3 (0.6)	0.1 (1.0)	0.0 (1.0)	0.6 (0.8)	0.3 (0.9)	0.3 (0.9)
- Range	[−1.7, 1.3]	[−1.4, 3.3]	[−1.7, 3.3]	[−1.5, 3.2]	[−1.3, 3.6]	[−1.2, 1.8]	[−1.7, 2.6]	[−1.6, 2.9]	[−0.9, 3.3]	[−1.5, 2.4]	[−1.7, 3.6]
- Missing	-	-	-	-	-	-	-	2 (1.4%)	2 (1.0%)	-	4 (0.1%)
**Protective behavioral strategies**											
** - Baseline**											
- Mean (*SD*)	0.9 (0.8)	0.5 (0.8)	0.5 (0.9)	0.4 (0.9)	0.4 (0.8)	−0.4 (0.9)	0.8 (1.2)	0.5 (0.8)	0.3 (0.9)	0.4 (0.8)	0.5 (0.9)
- Range	[−1.0, 3.1]	[−2.3, 2.9]	[−2.2, 3.4]	[−2.4, 3.2]	[−1.6, 2.6]	[−2.3, 2.7]	[−2.8, 3.2]	[−2.0, 3.4]	[−2.2, 3.4]	[−2.4, 2.6]	[−2.8, 3.4]
- Missing	18 (12.5%)	206 (24.9%)	258 (22.9%)	40 (16.4%)	-	-	66 (36.1%)	9 (6.4%)	-	72 (20.0%)	669 (18.1%)
** - Post-baseline**											
- Mean (*SD*)	1.0 (0.9)	0.6 (0.8)	0.6 (0.9)	0.6 (0.9)	0.4 (0.8)	−0.3 (0.9)	0.7 (1.1)	0.6 (1.2)	0.4 (0.9)	0.3 (0.9)	0.5 (0.9)
- Range	[−1.7, 3.4]	[−1.6, 3.2]	[−2.6, 3.5]	[−2.4, 2.6]	[−1.7, 2.9]	[−2.3, 1.6]	[−2.4, 2.8]	[−2.8, 3.4]	[−2.0, 3.4]	[−2.6, 2.6]	[−2.8, 3.5]
- Missing	0 (0.0%)	12 (1.5%)	25 (2.2%)	1 (0.4%)	6 (1.9%)	-	3 (1.6%)	2 (1.4%)	2 (1.0%)	-	51 (1.4%)

## Meta-analytic Mediation Model for Pretest–Posttest Designs

Clinical trials commonly use pretest–posttest designs in which participants are assessed at baseline and one or more follow-ups. In the current motivating data, half of the studies included a single follow-up within 12 months post-intervention (see Table 1). To accommodate the broadest range of follow-up schedules, we focus on evaluating mediation using longitudinal data from two time points: (1) baseline and (2) the first post-baseline follow-up for which both mediation and outcome data were collected in each study.

**Fig. 1:**
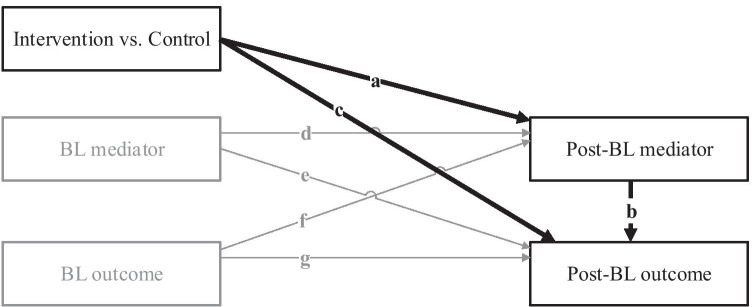
A two-wave longitudinal intervention mediation model. The key mediation-related paths are emphasized in black and the covariate paths are in gray. BL, baseline; post-BL, post-baseline

Figure 1 depicts a basic two-wave longitudinal mediation model (MacKinnon, 2008; Valente & MacKinnon, 2017) that controls for baseline levels of both the mediator and the study outcome. This is an extension of the classic cross-sectional mediation model outlined by Baron and Kenny (1986) that evaluates if (a) the intervention (vs. control) is prospectively associated with post-baseline improvements in the mediator, (b) post-baseline improvement in the mediator is associated with post-baseline improvements in the study outcome, and (c) the intervention (vs. control) is associated with the study outcome after controlling for the mediator (i.e., the direct effect). This mediation model can be easily extended to include additional treatment contrasts and covariates as well as to accommodate clustered data across multiple studies, within an SEM framework. Next, we describe the application of the basic two-wave longitudinal mediation model outlined in Fig. 1 to the Project INTEGRATE data. The meta-analytic mediation model consists of (1) an “overall model” that combines IPD across all studies and (2) “study-specific sub-models” that characterize potential differences between individual studies and inform the interpretation of the overall meta-analytic results.

### Overall Mediation Model

First, we detail the overall meta-analytic mediation model of the combined sample of all participants across all included studies. Let POST_PBS_*is*_ be the post-baseline PBS score of participant *i* in study *s*. Equation (1) is the first equation in the mediation model, which models the average, prospective association between each intervention group (vs. control) and post-baseline levels of the mediator variable, controlling for baseline levels of the mediator variable, PBS, and the study outcome variable, alcohol-related problems:

1 $${\mathrm{POST}\_\mathrm{PBS}}_{is}={b}_{0(A)}+{b}_{1(A)}{\mathrm{TX}\_\mathrm{MIPF}}_{is}+{b}_{2(A)}{\mathrm{TX}\_\mathrm{PF}}_{is}+{b}_{3(A)}{\mathrm{TX}\_\mathrm{GMI}}_{is}+{b}_{4(A)}{\mathrm{BL}\_\mathrm{ALCPROB}}_{is}+{b}_{5(A)}{\mathrm{BL}\_\mathrm{PBS}}_{is}+{b}_{6(A)}{\mathrm{MALE}}_{is}+{b}_{7(A)}{\mathrm{FIRSTYR}}_{is}+{b}_{8(A)}{\mathrm{NONWHITE}}_{is}+{e}_{is(A)},$$

where (*A*) identifies regression coefficients from the first of the two mediation model equations and ${e}_{is\left(A\right)}$ is a participant-specific residual error term. $\mathrm{TX\_MIPF}_{is}$, $\mathrm{TX\_PF}_{is}$, and $\mathrm{TX\_GMI}_{is}$ are dummy-coded variables that indicate random allocation to MI + PF, PF, or GMI, respectively (each coded 1), compared to controls (all coded 0). The regression coefficients ${b}_{1\left(A\right)}$, ${b}_{2\left(A\right)}$, and ${b}_{3\left(A\right)}$ quantify the covariate-adjusted average difference between participants who received (1) MI + PF, (2) stand-alone PF, or (3) GMI, respectively, compared to control participants. The covariate BL_PBS_*is*_ adjusts for initial levels of the PBS mediator, and the covariate BL_ALCPROB_*is*_ adjusts for initial levels of alcohol-related problems.

Let POST_ALCPROB_*is*_ be the post-baseline level of the study outcome variable, alcohol-related problems, of participant *i* in study *s*. Equation (2) is the second equation in the mediation model, which models the association between post-baseline levels of the mediator, PBS, and post-baseline levels of the study outcome, alcohol-related problems, adjusting for baseline levels of the mediator and study outcome variables:

2 $${\mathrm{POST}\_\mathrm{ALCPROBS}}_{is}={b}_{0(B)}+{b}_{1(B)}{\mathrm{TX}\_\mathrm{MIPF}}_{is}+{b}_{2(B)}{\mathrm{TX}\_\mathrm{PF}}_{is}+{b}_{3(B)}{\mathrm{TX}\_\mathrm{GMI}}_{is}+{b}_{4(B)}{\mathrm{BL}\_\mathrm{ALCPROB}}_{is}+{b}_{5(B)}{\mathrm{BL}\_\mathrm{PBS}}_{is}+{b}_{6(B)}{\mathrm{POST}\_\mathrm{PBS}}_{is}+{b}_{7(B)}{\mathrm{MALE}}_{is}+{b}_{8(B)}{\mathrm{FIRSTYR}}_{is}+{b}_{9(B)}{\mathrm{NONWHITE}}_{is}+{e}_{is(B)},$$

where (*B*) identifies regression coefficients associated with the second mediation model equation and ${e}_{is\left(B\right)}$ is a participant-specific residual error term. The regression coefficients ${b}_{1\left(B\right)}$, ${b}_{2\left(B\right)}$, and ${b}_{3\left(B\right)}$ provide the average *direct effect* of each intervention (vs. control) across studies on post-baseline levels of the study outcome variable. Both Eqs. (1) and (2) include the demographic covariates MALE_*is*_, FIRSTYR_*is*_, and NONWHITE_*is*_, which adjust for sex (1 = *men* vs. 0 = *women*), first-year student status (1 = *first-year* vs. 0 = *non-first-year*), and race (1 = *non-White* vs. 0 = *White*), respectively.

### Study-Specific Mediation Sub-models

Next, we describe the study-specific mediation sub-models, which inform the interpretation of the overall mediation model by characterizing variation in the results across studies. The mediation analysis is repeated separately and sequentially for each study by using sub-models of Eqs. (1) and (2) to include the estimable terms (i.e., evaluated intervention groups and demographic covariates with variability). For example, coefficients ${b}_{7\left(A\right)}$ and ${b}_{8\left(B\right)}$ are not estimable and hence excluded in the study-specific sub-models for studies 9, 16, and 22 because they recruited only first-year students. As an illustration, Eqs. (3) and (4) are the study-specific sub-models for study 22, which evaluated MI + PF vs. control:

3 $${\mathrm{POST}\_\mathrm{PBS}}_{i}={b}_{0\left(A\right)}+{b}_{1\left(A\right)}{\mathrm{TX}\_\mathrm{MIPF}}_{i}+{b}_{4\left(A\right)}{\mathrm{BL}\_\mathrm{ALCPROB}}_{i}+{b}_{5\left(A\right)}{\mathrm{BL}\_\mathrm{PBS}}_{i}+{b}_{6\left(A\right)}{\mathrm{MALE}}_{i}+{b}_{8\left(A\right)}{\text{NONWHITE}}_{i}+\begin{array}{c}{e}_{i\left(A\right)},\end{array}$$

4 $${\mathrm{POST}\_\mathrm{ALCPROB}}_{i}={b}_{0\left(B\right)}+{b}_{1\left(B\right)}{\mathrm{TX}\_\mathrm{MIPF}}_{i}+{b}_{4\left(B\right)}{\mathrm{BL}\_\mathrm{ALCPROB}}_{i}+{b}_{5\left(B\right)}{\mathrm{BL}\_\mathrm{PBS}}_{i}+{b}_{6\left(B\right)}{\mathrm{POST}\_\mathrm{PBS}}_{i}+{b}_{7\left(B\right)}{\mathrm{MALE}}_{i}+{b}_{9\left(B\right)}{\text{NONWHITE}}_{i}+\begin{array}{c}{e}_{i\left(B\right)},\end{array}$$

where (*A*) and (*B*) identify regression coefficients from the reduced first and second mediation model equations, respectively, *i* identifies the participant, and ${e}_{i\left(A\right)}$ and ${e}_{i\left(B\right)}$ are participant-specific residual error terms. For consistency, the subscripts in Eqs. (3) and (4) correspond with the same variables as those shown in the overall model Eqs. (1) and (2). As seen in Table 1, intervention groups not evaluated in a study become study-level missing data in the context of IPD meta-analysis. The parameters associated with missing treatment contrasts are excluded in the study-specific sub-model. Thus, ${b}_{2\left(A\right)}$, ${b}_{2\left(B\right)}$, ${b}_{3\left(A\right)}$, and ${b}_{3\left(B\right)}$ are excluded from Eqs. (3) and (4) since PF and GMI were not evaluated in study 22 by study design (i.e., ${\mathrm{TX}\_\mathrm{MIPF}}_{i}$ = 0 and ${\mathrm{TX}\_\mathrm{GMI}}_{i}$ = 0 for all participants *i* in study *s*), and ${b}_{7\left(A\right)}$, ${b}_{8\left(B\right)}$ are excluded by study design since all participants in study 22 were first-year students. It is important to note that the interpretation of each parameter estimate depends on the other parameters included in the model (see Jiao et al., 2020). However, if we assume that Eqs. (1) and (2) represent the true model for all studies, it is reasonable to assume the omitted coefficients in the sub-models are missing at random. In addition, since baseline PBS and alcohol-related problems are adjusted for in all sub-models, any interpretational bias associated with missing demographic covariates would be minimal.

## Accounting for Clustered Design Using SEM for Complex Survey Data

A key data feature of IPD combined from multiple studies is the nesting of individual participants within studies, which must be considered for accurate statistical inference (see also Mun et al., 2015, p. 36–38). To account for the nested data structure of IPD from multiple studies in a one-stage integrative analysis, parameter estimates and corresponding standard errors can be adjusted for clustering by utilizing either (1) a model-based approach using multilevel modeling that incorporates cluster-specific parameters (e.g., Huh et al., 2015, 2019) or (2) a design-based approach in which clustering is accommodated via complex survey analysis with weights applied to participants in a single-level analysis (e.g., Clarke et al., 2013, 2016; Li et al., 2020; Ray et al., 2014).

The advantage of design-based adjustment for clustering is that it can be implemented easily in an SEM framework and produces estimates that are comparable to multilevel modeling (Wu & Kwok, 2012), but with a lower computational burden. The computational efficiency of cluster-adjusted SEM makes it especially useful when combined with bootstrapping, the commonly accepted method for evaluating the statistical significance of the mediated (i.e., indirect) effect (see “Bootstrap Resampling with Multiple Imputation” later).

To evaluate the meta-analytic mediation model outlined in Eqs. (1)–(4), while accounting for the nested design of the data, we utilized SEM for complex survey data by first using the R package lavaan (Rosseel, 2012) to estimate an SEM that combines data across all studies in a single-level analysis followed by lavaan.survey (Oberski, 2014), which provides a design-based adjustment to account for clustering by study. SEM for complex survey data is analogous to the generalized estimating equation (Zeger et al., 1988) approach to analyzing multilevel data, which is also a design-based approach to accommodate clustered data. To account for widely varying sample sizes across studies, we weighted the data using the inverse of the square root of each study’s sample size as explained in Mun et al. (2015) and used in research applications (Clarke et al., 2013, 2016; Ray et al., 2014).

With respect to interpretation, the regression coefficients (i.e., fixed effects) produced by SEM for complex survey data are marginal estimates, which represent the average effects across all individuals. In contrast, regression coefficients estimated using a model-based approach are cluster-specific estimates that are conditional on specific values of the random effects (e.g., the deviation of a specific individual from the group average). When the outcome is modeled as normally distributed, regression coefficients produced by multilevel models (i.e., mixed-effects models) can be interpreted like marginal estimates, although this does not hold for extensions of multilevel modeling that use a non-identity link function, such as logistic or Poisson models (Atkins et al., 2013). Thus, the inference for a model that accounts for clustering using a design-based approach is functionally equivalent to multilevel modeling in the present application.

## Calculating the Indirect and Total Effect of Intervention

To calculate the *indirect effect* of each intervention type on the post-baseline study outcome via changes in the mediator, we calculate the product of the regression coefficients corresponding to (1) the association between intervention type and post-baseline PBS (i.e., ${b}_{1\left(A\right)}$, ${b}_{2\left(A\right)}$, and ${b}_{3\left(A\right)}$) and (2) the association between post-baseline PBS and changes in alcohol-related problems, ${b}_{6\left(B\right)}$. Equations (5)–(7) summarize the formulas used to calculate the indirect effects of MI + PF, stand-alone PF, and GMI vs. control, respectively, for the *overall* (Eqs. 1 and 2) and *study-specific* (Eqs. 3 and 4) models:

5 $$\text{MI+PF}:{b}_{1\left(A\right)}\times {b}_{6\left(B\right)},$$

6 $$\text{Stand-alone PF}:{b}_{2\left(A\right)}\times {b}_{6\left(B\right)},\text{ and}$$

7 $${\text{GMI}}:{b}_{3\left(A\right)}\times {b}_{6\left(B\right)}.$$

To calculate the *total effect* of each intervention type on post-baseline alcohol-related problems, we sum (a) the direct effect of each intervention type on alcohol-related problems (i.e., ${b}_{1\left(B\right)}$, ${b}_{2\left(B\right)}$, and ${b}_{3\left(B\right)}$) from Eq. (2) and (b) the corresponding indirect effect of each intervention type calculated in Eqs. (5), (6), or (7). Equations (8)–(10) summarize the formulas used to calculate the total effects of MI + PF, stand-alone PF, and GMI vs. control, respectively, for the *overall* (Eqs. 1 and 2) and *study-specific* (Eqs. 3 and 4) models:

8 $$\text{MI+PF}:{b}_{1\left(B\right)}+\left({b}_{1\left(A\right)}\times {b}_{6\left(B\right)}\right),$$

9 $$\text{Stand-alone PF}:{b}_{2\left(B\right)}+\left({b}_{2\left(A\right)}\times {b}_{6\left(B\right)}\right),\text{ and}$$

10 $$\text{GMI}:b_{3(B)} + (b_{3(A)} \times b_{6(B)}).$$

## Bootstrap Resampling with Multiple Imputation

To evaluate the magnitude and statistical significance of the estimates from the mediation model, including regression coefficients, indirect effects, total effects, and *R*^2^ values, we used bootstrap resampling (Efron & Tibshirani, 1993) in which the mediation analyses are replicated across 5000 bootstrapped data sets to calculate the mean point estimate and 95% confidence interval for each parameter. Bootstrap estimation involves random sampling of observations with replacement from the original data set such that the sample is treated as if it were the population. The effect of sampling with replacement is that an observation may be represented more than once, whereas some observations may be left out in any given bootstrap sample. As a result, the bootstrap sample is equal in size to the original but is not identical.

Because missing data present in the original data set will also be reflected in the bootstrap data set, an additional consideration is needed to handle missing data when bootstrapping. In the context of an IPD meta-analysis, there can be two sources of missing data: (1) study-level missing data due to a variable not being assessed or without variation (see Jiao et al., 2020; Kim et al., 2014) and (2) participant-level missing data due to nonresponse. In the context of the Project INTEGRATE data, study-level missing data occurred because only one study evaluated all three intervention groups, the rest evaluated a subset of intervention groups (i.e., one or two), and also because some studies exclusively targeted first-year students or women. These are not missing variables within the original studies; however, in the context of meta-analysis, they are missing or inestimable covariates at the study level. As described previously, we excluded the corresponding treatment contrast or demographic covariate from the corresponding study-specific mediation sub-model. Therefore, study-level missing variables were not imputed.

As seen in Table 2, there were also participant-level missing variables. Thus, to minimize bias in the results of the mediation analysis due to missing mediator, outcome, and/or covariate data, bootstrapping was combined with multiple imputation. Multiple imputation is a widely used method for accommodating missing data. Furthermore, simulation research supports combining multiple imputation with bootstrapping (Little & Rubin, 2002; Schomaker & Heumann, 2018). There are several ways to combine multiple imputation and bootstrapping, each with pros and cons (Brand et al., 2019). In the present study, we chose to bootstrap first, followed by multiple imputation, which is more computationally intensive but produces confidence intervals that more accurately reflect uncertainty due to missing data (Bartlett & Hughes, 2020).

First, a stratified bootstrap was performed in which participants, including those with missing data, were randomly sampled with replacement separately by study and intervention group then combined into a single bootstrapped data set of equal size to the original data set. The stratification by study and intervention group accounted for the clustered design (i.e., participants nested within studies and groups) and maintained consistent sample sizes in subsequent analyses, within and across studies, as well as across all intervention groups. A total of 5000 bootstrap-resampled data sets were generated. Second, for each of the bootstrap-resampled data sets, a set of ten imputed data sets were generated via multivariate normal imputation with the R package Amelia (Honaker et al., 2011). According to simulation findings by Bartlett and Hughes (2020), ten imputations per bootstrap replicate provide approximately accurate confidence intervals when multiple imputation is nested within bootstrapping.

The mediation analysis was repeated for each multiply imputed data set, and the results were combined across ten imputed data sets. This yielded a set of 5000 estimates for each parameter in the mediation model, one for each bootstrap replicate. The collection of bootstrap estimates approximates the sampling distribution for each parameter and accommodates non-normally distributed estimates, such as the indirect and total effects. The point estimate for each parameter was calculated as the mean across the 5000 bootstrap replications. Bias-corrected and accelerated 95% confidence intervals were calculated to assess the indirect and total effects, as recommended by MacKinnon et al. (2004).

## Analysis of the Motivating Data and the Summary of Findings

Annotated computer code in R for fitting the model, along with example data, can be accessed in the online repository (10.17632/t2yk5kt3bw.1; Huh et al., 2021).

**Fig. 2:**
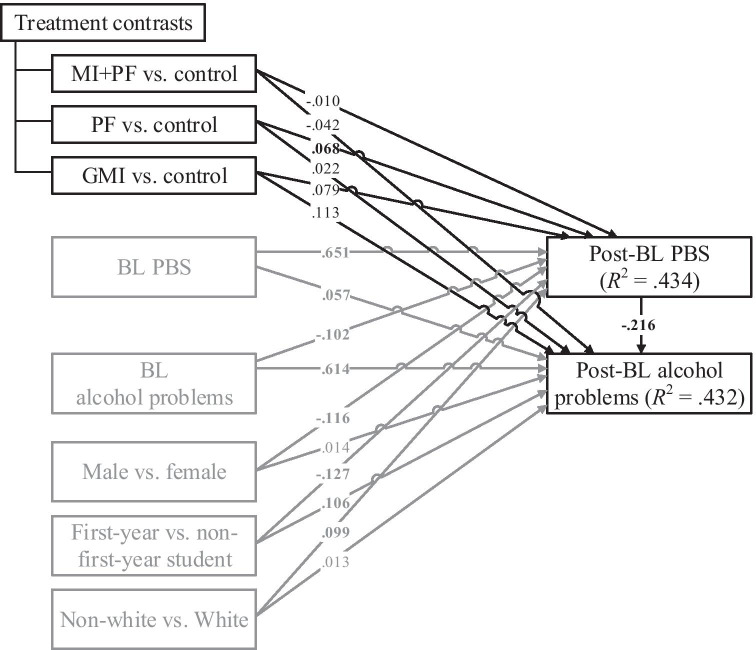
Overall mediation model evaluating change in protective behavioral strategies as a pathway by which brief motivational intervention improves alcohol-related problems for college students who drink. The key mediation-related paths are emphasized in black and the covariate paths are in gray. All path coefficients are standardized betas (with respect to the outcomes only), and results highlighted in bold are statistically significant (*p* < .05). MI + PF, individually delivered motivational interviewing with personalized feedback; PF, stand-alone personalized feedback; GMI, group motivational interviewing; BL, baseline; post-BL, post-baseline; PBS, protective behavioral strategies

Figure 2 is a path diagram that summarizes the estimated associations from the overall mediation model of the combined sample. The path coefficients are standardized with respect to the outcome, which can be interpreted as the effect that a unit difference in each predictor has on the corresponding outcome variable, holding all other covariates constant. For treatment contrasts and other indicator variables, the path coefficients correspond with the difference between groups (e.g., MI + PF vs. control) in *SD*s of the outcome. For continuous predictors (i.e., alcohol-related problems, PBS), the standardized coefficient can be interpreted as the change in *SD*s of the outcome for a unit difference in the predictor. The overall mediation model explained 43% of the variance in both post-baseline PBS and post-baseline alcohol-related problems.

The paths of interest are (1) the prospective association between each intervention and post-baseline levels of the mediator (PBS) and (2) the association of the mediator and the outcome at post-baseline. Of the three interventions, only stand-alone PF had a statistically significant association with the mediator, with a .07 *SD* increase (95% CI = [.01, .12]) in post-baseline PBS as compared to control. A one-*SD* increase in post-baseline PBS, in turn, was associated with a .22 *SD* reduction (95% CI = [−.26, −.17]) in post-baseline alcohol-related problems.

**Fig. 3:**
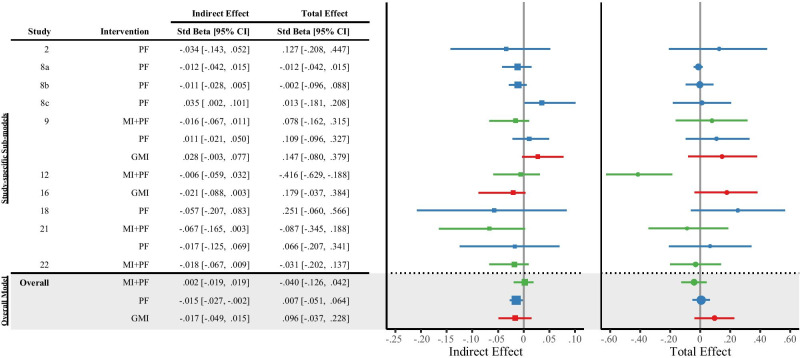
Forest plot of the indirect and total effect of brief motivational interventions on alcohol-related problems via protective behavioral strategies. The study-specific sub-model results (top portion) illustrate the heterogeneity in the indirect and total effects of intervention across the ten studies. The overall model results (bottom portion, highlighted in gray) for the combined sample summarize the *indirect *and* total effects* of each intervention type on post-baseline alcohol-related problems. Std Beta, standardized beta, with respect to the outcome only; CI, confidence interval; MI + PF, individually delivered motivational interviewing with personalized feedback (green-colored estimates); PF, stand-alone personalized feedback (blue-colored estimates); GMI, group motivational interviewing (red-colored estimates)

Figure 3 is a forest plot that summarizes the key mediation-related results (i.e., indirect and total effects) from (a) the ten study-specific sub-models (top portion) and (b) the overall model (bottom portion, highlighted in gray) of the combined sample. A negative coefficient can be interpreted as a prospective improvement (i.e., reduction) in alcohol-related problems at post-baseline. Stand-alone PF, compared with control, was associated with a statistically significant, albeit small, reduction in alcohol-related problems via increased use of PBS (*β* =  −.01, 95% CI = [−.03, −.002]). Neither MI + PF nor GMI was associated with statistically significant reductions in alcohol-related problems, compared with control, through improvements in PBS.

An additional sensitivity analysis was conducted to evaluate the consistency of the findings when the mediation analysis was repeated by leaving out one study at a time, sequentially (see the Supplemental Material for a summary). The indirect and total effects of each intervention approach were consistent across the sensitivity models, suggesting that the results were robust and not driven by any single influential study.

## Discussion

The literature evaluating mechanisms of intervention effect has relied almost exclusively on single-study intervention trials, which are frequently underpowered to evaluate mediation hypotheses (Fritz et al., 2015). This methodological illustration details a meta-analytic mediation analysis approach that leverages IPD across multiple studies to evaluate mechanisms of change longitudinally. Specifically, the approach evaluates whether the prospective change in a mediator following intervention is accompanied by a change in the outcome. Moreover, the approach can accommodate missing data commonly encountered in clinical trial data, making it a practical option for meta-analytic mediation analysis.

The illustrated SEM approach combines well-established quantitative methodologies, including SEM with design-based adjustment for clustering, bootstrap estimation of mediated effects, and multiple imputation, to test mediation with accuracy and precision. We describe how to calculate the magnitude of a mediated effect within and across studies and assess its statistical significance in a way that (a) accounts for the clustering of participants within the study, (b) uses all available data, and (c) produces point estimates and confidence intervals for the indirect and total effects of an intervention that account for the non-normal distribution that arises from a product of coefficients.

At a substantive level, it is of interest that greater use of PBS mediated the effect of stand-alone PF intervention on alcohol-related problems. Specifically, participants receiving stand-alone PF had greater improvement in PBS utilization compared with participants randomized to the control comparison. Greater PBS utilization, in turn, was associated with concurrent reductions in alcohol-related problems. Although statistically significant, it is important to note that the mediated effect of PF via a change in PBS was quite small, equivalent to a .01 *SD* difference in the reduction in alcohol-related problems. The small mediated effect may be because brief motivational interventions, including PF, do not increase the use of PBS substantially. However, the results from this study may suggest that stand-alone PF focusing on a few salient points, such as PBS, may be more likely to induce behavior change than formats that use multiple modalities (Ray et al., 2014).

Although the effect of brief motivational interventions on alcohol-related problems via a change in PBS appeared to be quite small in the present study, our findings are consistent with the evidence of some PBS-based interventions failing to improve outcomes (Martens et al., 2013). In addition, college students utilize PBS for different reasons, with some students engaging in PBS to get intoxicated faster while trying to prevent the most extreme harm. Therefore, the increased use of PBS can increase alcohol-related problems for some students unmotivated to change their drinking, while low-risk drinkers may use them to effectively limit harm from drinking (Li et al., 2020). The average effect that we focused on in the current study, although important, needs to be examined further for heterogeneous mediational paths, accounting for students’ different motivations for drinking and PBS use.

It is important to note that most of the studies evaluated only one or two intervention groups and not all three interventions. The unbalanced nature of the intervention groups across studies is a typical challenge in a meta-analysis across heterogeneous studies, including IPD data syntheses (Brincks et al., 2018; Huh et al., 2019), and can complicate the interpretation of findings. However, the motivating data featured a large, pooled sample of college students from brief motivational intervention studies, which permitted more robust mediation estimates for all the intervention types (i.e., MI + PF, stand-alone PF, and GMI) than would be possible in individual trials. Furthermore, we previously developed commensurate measures across trials for key constructs and carefully controlled for baseline levels of both the mediator and outcome variables, which bolsters confidence in the findings.

An important advantage of meta-analytic mediation analysis using IPD compared to traditional meta-analysis is the ability to evaluate the prospective association between baseline participant characteristics and change in PBS, which yielded additional insights. As seen in Fig. 2, we found that men (vs. women), first-year students (vs. non-first-year students), White students (vs. non-White students), and those with more severe alcohol-related problems at baseline showed less improvement in PBS use at a follow-up. The ability to make inferences regarding participant-level change shows the benefit of this IPD-based approach for evaluating mechanisms of change in prevention research.

### Limitations and Future Directions

It is important to consider the limitations of the present study. First, we could not evaluate if change in the mediator *preceded* change in the study outcome, which would require data from at least three time points. Second, the approach relies on assumptions about missing data that we believe to be reasonable, including that the absence of an intervention group in a study does not bias the overall findings. However, further investigation via simulation study may be needed to identify potential areas of improvement. Third, this methodological illustration focuses on evaluating a single mediator; however, the approach we detailed can be extended to models with multiple mediators. Fourth, a minor drawback to our approach is that combining multiple imputation with bootstrapping is computationally intensive; however, the estimation times (e.g., 10–20 min per model) encountered in the present study are feasible for applied research. Finally, our motivating example focused on a relatively normally distributed mediator variable and outcome of interest. Future research might examine extensions of this approach within a generalized SEM framework to binary, count, or other outcome distributions.

### Conclusions

The SEM approach detailed in this methodological illustration is a flexible approach for conducting a mediation analysis that leverages the most granular information from multiple studies and overcomes key challenges that arise when combining clinical trial data. The annotated R code and data provide additional guidance for researchers who wish to apply the method in their own research, and we hope it will motivate further development in meta-analytic mediation methodology and its applications in prevention science.

## Supplementary Information

Below is the link to the electronic supplementary material.

Supplementary file1 (DOCX 246 KB)
