# Supplementary material for: A Structural Equation Modeling Approach to Meta-analytic Mediation Analysis Using Individual Participant Data: Testing Protective Behavioral Strategies as a Mediator of Brief Motivational Intervention Effects on Alcohol-Related Problems
Source: Prev Sci. 2021 Nov 12;23(3):390–402. doi: 10.1007/s11121-021-01318-4 (PMC8975788; doi:10.1007/s11121-021-01318-4)
Supplement: Supplementary file 1 — Supplementary file1 (DOCX 246 KB) [file 11121_2021_1318_MOESM1_ESM.docx]

**
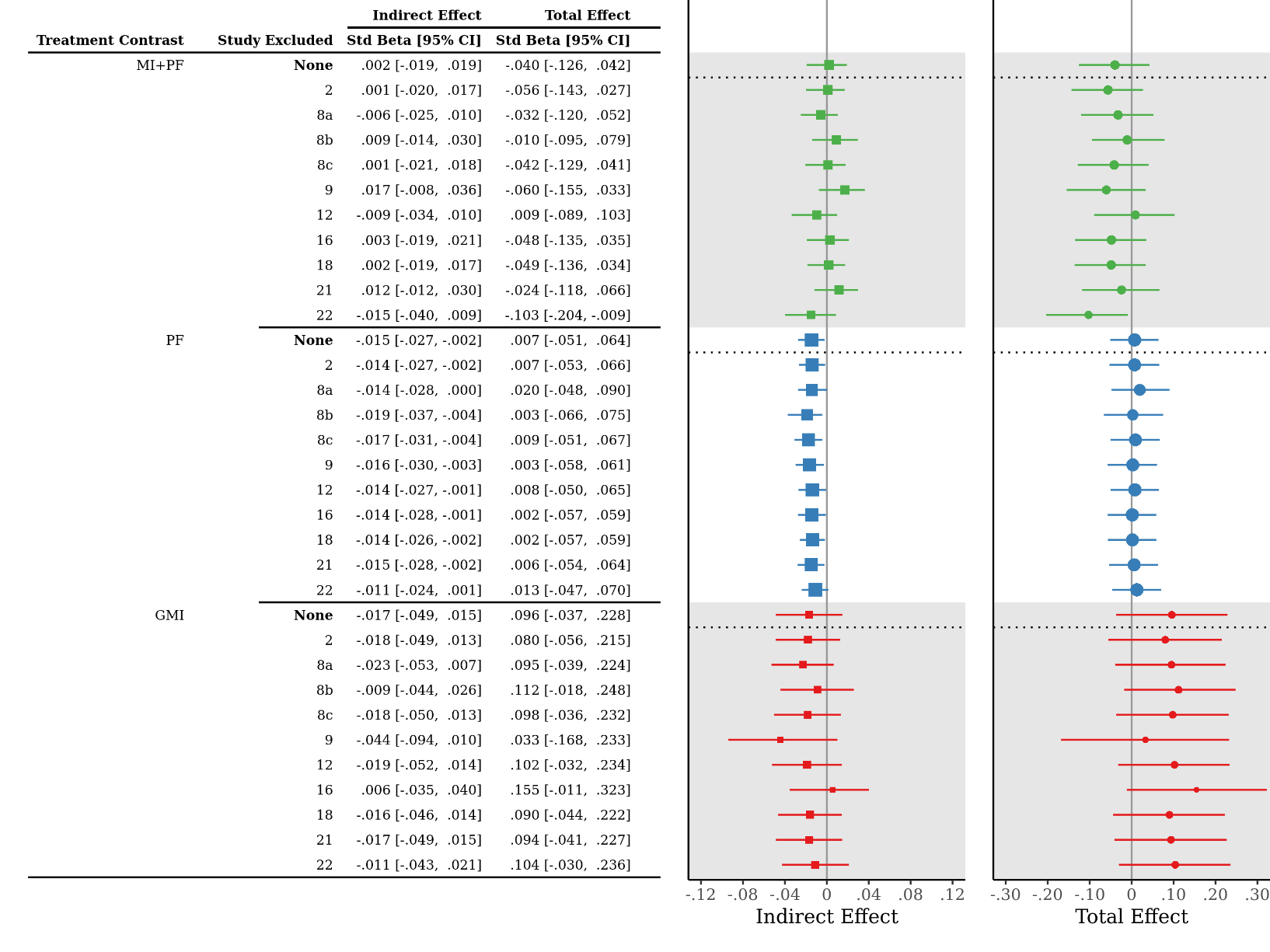
**

**Supplemental Figure.** Results of the leave-one-out cross-validation analysis. Std Beta = Standardized Beta, with respect to the outcome only; CI = Confidence Interval; MI+PF = Individually delivered Motivational Interviewing with Personalized Feedback (green-colored estimates); PF = Stand-alone Personalized Feedback (blue-colored estimates); GMI = Group Motivational Interviewing (red-colored estimates).
